# Meta-Analysis of Serum Insulin-Like Growth Factor 1 in Alzheimer’s Disease

**DOI:** 10.1371/journal.pone.0155733

**Published:** 2016-05-26

**Authors:** Philip P. Ostrowski, Andrew Barszczyk, Julia Forstenpointner, Wenhua Zheng, Zhong-Ping Feng

**Affiliations:** 1 Department of Physiology, University of Toronto, Toronto, Ontario, Canada; 2 Faculty of Health Science, University of Macau, Macau, China; Torrey Pines Institute for Molecular Studies, UNITED STATES

## Abstract

Insulin-like growth factor 1 (IGF-1) serum levels have been reported to be altered in Alzheimer’s disease patients, and it was suggested that the changes in IGF-1 serum level may play a role in disease pathology and progression. However, this notion remained controversial due to conflicting findings. We conducted a meta-analysis to determine the relationship between IGF-1 serum levels and Alzheimer’s disease. We searched the databases PUBMED, Ovid SP, and Cochrane library for relevant studies. The primary data analyzed was serum IGF-1 from Alzheimer’s disease subjects and controls. Pooled weighted mean difference using a random effects model was used to determine the relationship between serum levels and disease state. Nine studies were included in the meta-analysis compromising a total of 1639 subjects. The pooled weighted mean difference was -2.27ng/ml (95% CI: [-22.221, 17.66]) with a P value of 0.82. Thus our finding did not show clear relationship between low IGF-1 and Alzheimer’s disease subjects. We did not find evidence of publication bias by analyzing a funnel plot as well as Egger’s and Begg’s tests. While eight out of the nine studies included in this meta-analysis detected a statistically significant increase or decrease in serum levels of IGF-1 in Alzheimer’s disease subjects, the analysis as a whole did not show a significant trend in either direction. Thus, IGF-1 level is likely a critical personalized factor. A large database of clinical trials is required for better understanding the relationship between IGF-1 levels and Alzheimer’s disease.

## Introduction

Insulin-like growth factor 1 (IGF-1) is a 7.5kDa peptide hormone produced primarily in the liver and also in smaller quantities in other organs such as the brain [1]. IGF-1 production in the liver is regulated by growth hormone secreted by the pituitary gland, while regulation in other tissues is not yet fully understood. In serum, IGF-1 binds to a family of insulin-like growth factor binding proteins (IGBPs) that extend its serum half-life. The primary target of IGF-1 is the IGF-1 receptor (IGF-1R), but can also activate the insulin receptor [2]. Downstream targets of IGF-1R include activation of the MAPK/ERK and PI3K/AKT pathways, which results in pro-growth and anti-apoptotic signals [3]. IGF-1 levels are high at a young age and then slowly decrease until death [4]. Excess IGF-1 can result in acromegaly, a condition characterized by excessive growth, while lack of IGF-1 can result in dwarfism [5, 6]. Increased IGF-1 is also linked to a high risk for certain cancers, likely due to enhancement of cell proliferation [7]. IGF-1 serum levels are reduced in diabetes [8].

IGF-1 plays an important role in neurogenesis and neurodevelopment, and abundant IGF-1 receptors are expressed in the brain [9]. The majority of IGF-1 in the brain is thought to be transported from serum across the blood brain barrier with the aid of megalin/LRP2 and LRP1 [10, 11]. IGF-1 binds to megalin/LRP2 on the endothelial cell surface and is transported across the cell and in turn blood brain barrier via transcytosis [12]. IGF-1 import can also be facilitated by LRP1, which is regulated by neuronal activity [11]. Hippocampal IGF-1 levels are positively correlated with serum IGF-1 levels and in otherwise healthy rats increasing the level of the latter will result in an increase in the former [13]. Brain IGF-1 is important for cognitive function and may stimulate neurogenesis [14]. Glucose metabolism in the brain is also regulated by IGF-1 and IGF-1R, and a reduction of signaling in this pathway decreases GLUT4 expression and glucose utilization [15–17]. Low serum levels caused by rare mutations in the IGF-1 gene lead to declined cognitive abilities that can be restored by supplementation with recombinant IGF-1 [18]. IGF-1 levels also decrease with age [19] and in diabetes [20], both coinciding with declined cognitive abilities.

IGF-1 regulates the signaling pathways that are altered in Alzheimer’s disease (AD). For instance IGF-1 enhances the survival of neurons that have been exposed to beta amyloid and inhibits tau phosphorylation through the inhibition of GSK-3β [21–25]. Furthermore, the IGF-1 pathway is dysregulated in AD, with alterations in both the levels and phosphorylation state of IGF-1R as well as the levels of IGF-1 and IGF-1R mRNA in the brain [26]. This dysregulation appears to be progressive, becoming more severe as the disease continues.

Animal models of AD have been used to study the relationship between IGF-1 and AD *in vivo*. These animals models include transgenic mice which have mutant amyloid precursor protein (APP) and presenilin 1 (PS1), both of which are associated with familial early onset AD in humans, or mutant tau protein that is not normally associated with familial AD in humans but results in a human-like AD pathology [27], and mice treated with aluminum chloride (AlCl_3_) or streptozotocin to induce AD-like pathologies [28, 29]. *In vivo* animal studies suggest that IGF-1 is an important mediator in the clearance and regulation of beta amyloid in the brain [30]. Systemic IGF-1 infusion in transgenic mice with mutant APP and presenilin led to export of beta amyloid into the serum and reduction of brain beta amyloid levels [31]. Furthermore, IGF-1R blockade in the choroid plexus results in a buildup of beta amyloid in the brain and AD-like pathology [23]. In wild type mice with beta amyloid infused into the brain, the systemic addition of IGF-1 was found to lower the toxicity of beta amyloid, further demonstrating that systemic administration of IGF-1 is neuroprotective [32]. IGF-1 deficiency caused earlier plaque formation in a transgenic mouse model of AD [33]. In contrast, independent studies showed that decreasing IGF-1R activity in the brain improved AD pathology in Igf1r+/- mice [34], indecated by densely packed aggregates which conferred decreased toxicity. However, decreasing IGF-1R activity in the brain did not prevent beta-amyloid production [34], This is likely due to beta-amyloid toxicity being greatest when in soluble oligomers versus in plaques/aggregates [35]. Consistent to these findings [34], studies using AD-like (APP/PS1) mouse knockout models of IGF-R and IRS-2, a signaling protein downstream of IGF-1R, also present with decreased toxicity even when plasma beta-amyloid levels increased [36, 37]. The AD-like animals lacking IGF-1R showed improvement in spatial memory with reduction of anxiety in the adulthood [37]. In addition, lowering serum IGF-1 via protein restriction diet ameliorated AD pathology in transgenic mouse models [38]. Most surprisingly, the administration of IGF-1 into the serum failed to alter beta amyloid levels in multiple compartments across transgenic rats, mice, and dogs [39]. These observations collectively questioned the use of IGF-1 as a treatment for AD, and challenged the notion that increased serum IGF-1 is neuroprotective in AD.

To better understand the potential role of IGF-1 in AD, in this study we compared the plasma, brain, and CSF levels of IGF-1 across multiple studies involving animal models of AD and analyzed human studies pertaining to the relationship between IGF-1 levels and AD. The goal of this study is to provide an unbiased and comprehensive analysis of the relationship between serum IGF-1 and AD in humans.

## Methods

To find relevant studies, the authors (P.O., J.F., A.B, and Z.P.F.) searched PUBMED, Ovid SP, and Cochrane library. The keyword combinations used in all three databases were “Alzheimer AND insulin like growth factor”, “Dementia AND insulin like growth factor”, and “Cognitive disorders AND insulin like growth factor”. Databases were searched on August 13^th^ 2014 with no language restrictions. No data was found when searching for unpublished studies. Inclusion criteria were as follows: 1) AD diagnosis using defined criteria; 2) matched control group; 3) serum IGF-1 values reported for both groups. Only primary studies reporting novel data were included in this study.

From the selected studies we extracted the authors’ names, publication year, study size, sample sizes, mean serum IGF-1 levels, mean age, serum collection protocol, serum analysis protocol, AD diagnosis criteria, as well as IGFBP-3 and CSF IGF-1 levels, if available. Serum IGF-1 values were analyzed in ng/mL, and were converted from nM using a molecular weight of 7.5 kDa [40], if required. For data reported as standard error of the mean, the equation $SD\;=\;SEM\;\times \;\sqrt{n}$ was used to convert the data to standard deviation which was used in this study. Serum IGF-1 was analyzed using Review Manager (RevMan; Version 5.3. Copenhagen: The Nordic Cochrane Centre, The Cochrane Collaboration, 2014). Studies were analyzed using the weighted mean difference between AD and control groups within a study, and a random effects model on pooled mean differences to determine statistical significance across all included studies, which was indicated by a *P* value of less than 0.05. Heterogeneity of the studies was determined using Review Manager I^2^ statistics, while publication bias was determined using Egger’s and Begg’s tests. Grubb’s test was used to determine whether any studies were outliers.

## Results

We first compared the plasma, brain, and CSF levels of IGF-1 across multiple studies involving animal models of AD. Table 1 shows that brain and/or CSF IGF-1 levels decreased in APP and APP/PS1 transgenic mice as well as AlCl_3_-treated mice [28, 41, 42]. Interestingly, serum IGF-1 is increased in APP, APP/PS1, and 3xTG transgenic mouse models [38, 42]. These findings suggest that there is altered IGF-1 transport between the CSF and serum in transgenic mouse models, as the ratio of CSF/serum IGF-1 is lower than in wild type controls [38]. If this is the case, then the elevation of serum IGF-1 levels may not in turn elevate CSF IGF-1 to a significant degree.

**Table 1:** Summary of the reported IGF-1 levels of serun and CSF/brain from AD or wildtype mice.

First Author (Year)	Journal	Animal	Model	AD Serum IGF-1 (ng/ml)	CSF/Brain IGF-1 (ng/ml)
**Trueba-Saiz, A (2013)** [42]	Translational Psychiatry	Mouse	APP	↑	↓
			APP/PS1	↑	↓
**Parrella, E (2013)** [38]	Aging Cell	Mouse	3xTG	↑	-
**Hu, Y (2013)** [41]	Neuroscience Bulletin	Mouse	APP/PS1	-	↓
**Fadl, N (2013)** [28]	Hum Exp Toxicol	Mouse	AlCl3	-	↓

**Fig 1:**
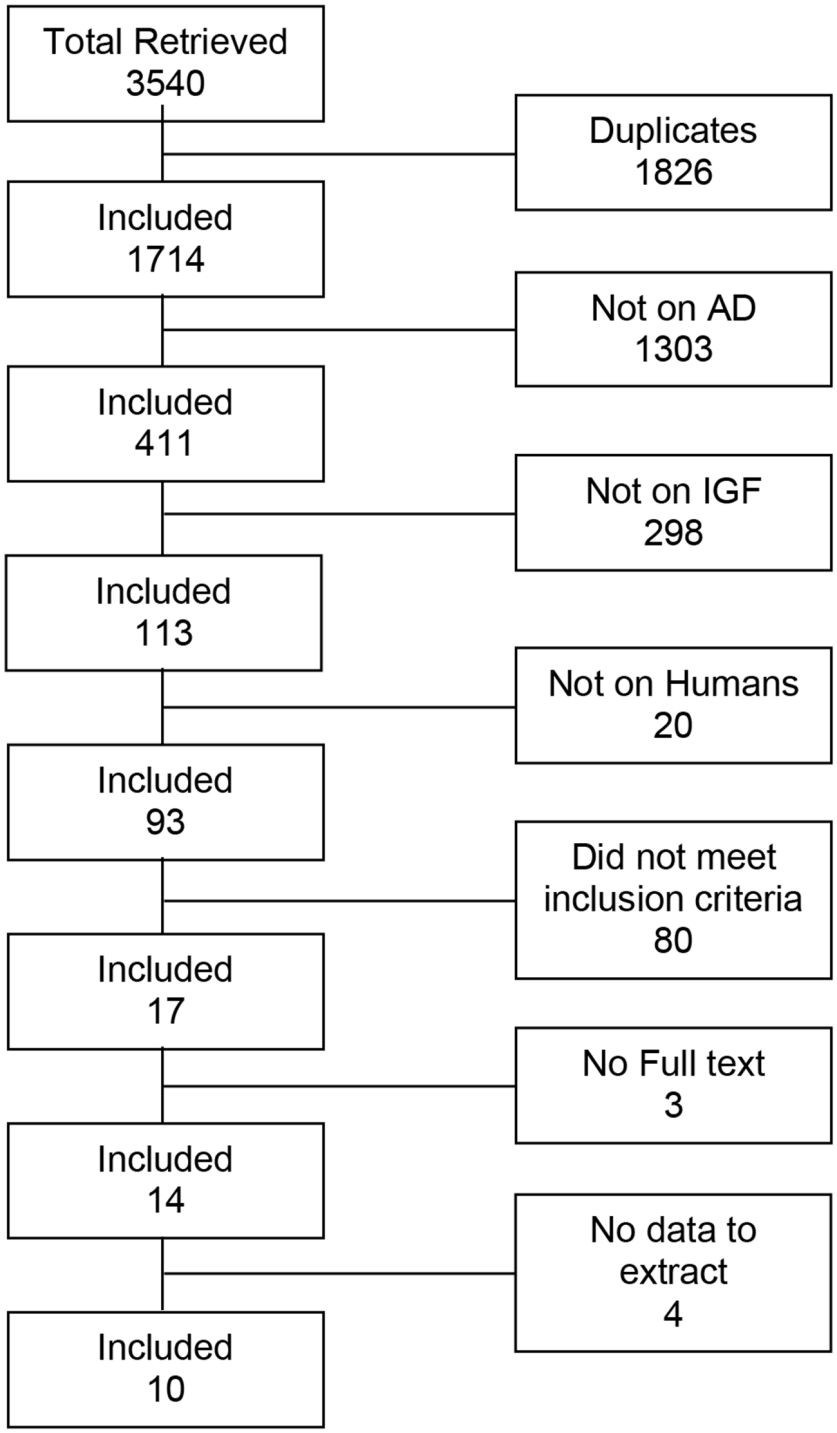
Study selection process (PRISMA flowchart).

To better understand whether IGF-1 is a protective agent in AD, we identified a total of 3540 studies from the database search, yielding 10 studies that provided serum IGF-1 values that could be used for analysis (Fig 1). We were unable to extract data from four relevant studies in addition to those 10, and another two studies which analyzed the incidence of AD in relation to serum IGF-1 but did not report serum levels for AD patients. Serum IGF-1 was analyzed by either radioimmunoassay or ELISA. Among the included 10 studies there were 850 AD patients and 871 controls for a total of 1721 participants, ranging from just 15 participants in the smallest study to 437 in the largest. Subject AD diagnosis in included studies was made by a combination of DSM-3R, DSM-4, DSM-5, and NINCDS-ADRDA guidelines. Disease progression was not reported by any of the included studies. The populations of study subjects did not overlap. A summary of included studies is presented in Table 2. The study by Duron et al reported male and female results separately; the means, standard deviation, and sample size were combined, and the combined standard deviation was calculated using equation 5.38 from *Statistical Simulation*: *Power Method Polynomials and other Transformations* [43].

**Table 2:** Summary of the studies that are included in this analysis.

First Author (Year)	Journal	Subjects (N)	Serum IGF-1 (ng/ml)	CSF IGF-1 (ng/ml)
**Alvarez, A. (2007)**[50]	Neurobiol Aging	171	IGF-1 ↓ in AD	-
**Duron, E. (2012)**[48]	J Clin Endocrinol Metab	437	IGF-1 ↓ in AD men	-
**Ghigo, E. (1993)**[44]	Dement Geriatr Cogn	29	IGF-1 ↑ in AD	-
**Hertze, J. (2014)**[49]	Bmc Neurology	164	No Change	No Change
**Mustafa, A. (1999)**[51]	Dement Geriatr Cogn	15	IGF-1 ↓ in AD	-
**Salehi, Z. (2008)**[45]	Biofactors	82	IGF-1 ↑ in AD	IGF-1 ↑ in AD
**Trueba-Saiz, A. (2013)**[42]	Translational Psychiatry	45	IGF-1 ↑ in AD	IGF-1 ↓ in AD
**Vardy, E. (2007)**[46]	J Alzheimers Dis	213	IGF-1 ↑ in AD	-
**Vargas, T. (2011)**[47]	Neurobiol Aging	232	IGF-1 ↑ in AD	-
**Watanabe, T. (2005)**[52]	J Am Geriatr Soc	333	IGF-1 ↓ in AD	-

**Fig 2:**
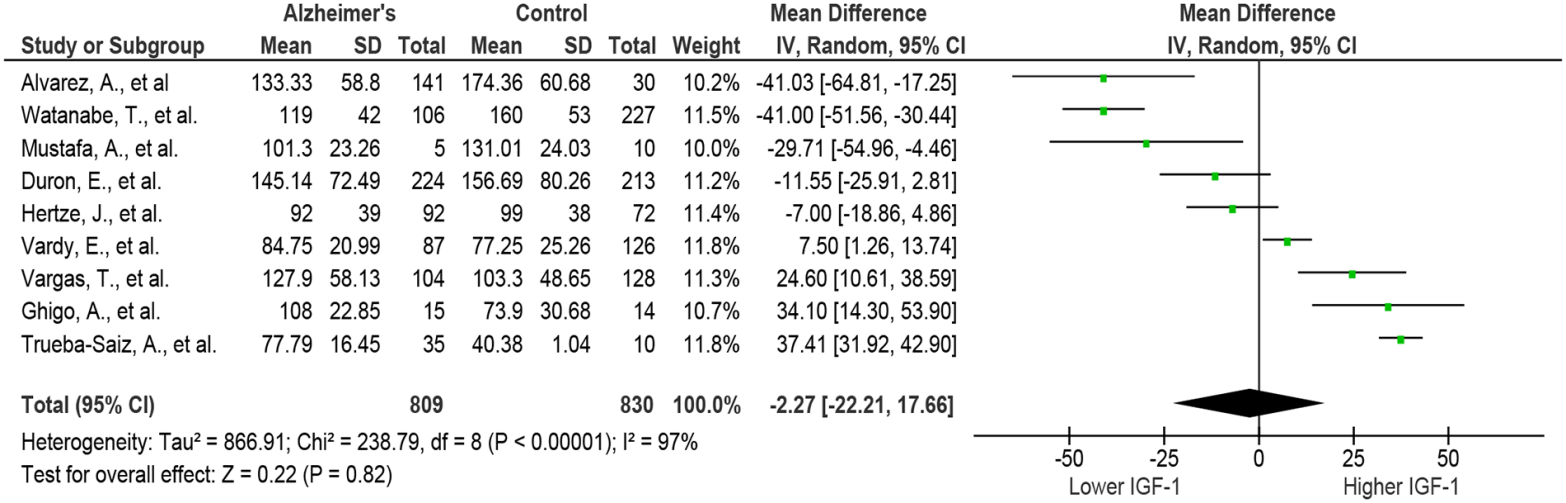
Forest plot of serum IGF-1 in AD and control subjects in included studies. The analysis did not include the outlier study by Salehi et al. The analysis showed that the difference is not significant within the groups in whole. CI: confidence intervals.

**Fig 3:**
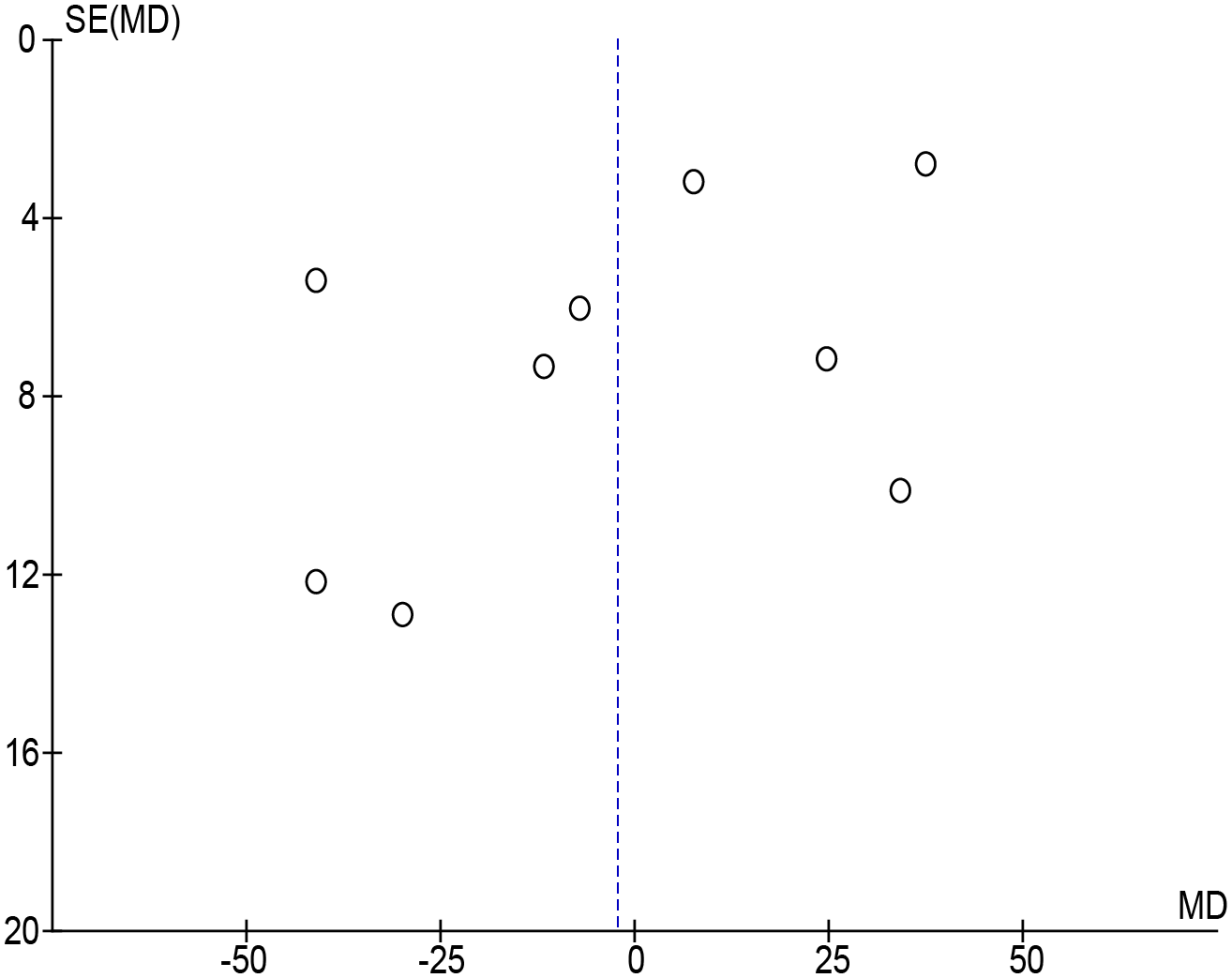
Funnel plot of serum IGF-1 in AD and controls in included studies. Funnel plot for all 9 studies included trials to visualize potential publication bias. The shape of funnel plots did not reveal obvious evidence of asymmetry, suggesting no publication bias.

From the 10 studies included, 5 reported [42, 44–47] that AD subjects had significantly higher serum IGF-1 levels, 2 reported [48, 49] no significant difference between the two groups, and 3 reported [50–52] lower serum levels of IGF-1. The study by Duron et al found no difference between control and AD, however men with AD had significantly lower IGF-1 when subjects were separated by sex [48]. We also noticed that the serum IGF-1 values reported by Salehi et al [45] were much greater than those in other studies for AD subjects or than those expected for the age group being tested [53]. When the data from Salehi et al were included in the analysis, it led to a pooled weighted mean difference with a random effects model of 20.58 ng/mL (95%CI: [-5.17, 46.32]; I^2^ = 98%; Cochran’s Q = 1433.16; P<0.00001) in favor of greater IGF-1 in AD, however there was no overall effect of Z = 1.57; p = 0.12. Using the Grubb’s test we determined that the results from Salehi et al [45] were an outlier (P<0.00001), and re-analyzed the remaining studies without this data. The remaining 9 studies which were composed of 809 AD subjects and 830 control subjects for a total of 1639. The pooled weighted mean difference using a random effects model was then -2.27 ng/mL (95%CI: [-22.21, 17.66]; I^2^ = 97%; Cochran’s Q = 866.91; P<0.00001) in favor of lower IGF-1, and the overall test for effect was Z = 0.22; p = 0.84 (Fig 2). These results suggest that AD subjects have almost identical serum IGF-1 levels to matched controls. We then conducted a funnel plot of the data and employed Egger’s (p = 0.32) and Begg’s (p = 0.22) tests, and found no evidence of publication bias (Fig 3).

Table 3 shows the summary of the studies which investigated IGF-1 and AD but were not included because these studies did not provide values of serum IGF-1 levels that are required for the analysis. Two cohort studies [54, 55] did not report serum IGF-1 levels and had opposite conclusions on the effects of serum IGF-1 and risk of AD. An additional four studies measured serum IGF-1 levels, three of which concluded that AD subjects had greater serum IGF-1 [40, 56, 57], and one which concluded that AD subjects had lower serum IGF-1 [58]. The dichotomy of results from both included and omitted studies of serum IGF-1 in AD has led some researchers to measure other aspects of the IGF-1 pathway, including CSF IGF-1, CSF/serum IGF-1 ratio, and IGFBP-3 levels. However there does not appear to be a clear trend in any of these parameters, and the limited number of studies reporting these measures precludes statistical analysis.

**Table 3:** Summary of the studies which investigated IGF-1 and AD but are not included in this analysis because these studies did not provide actual values of serum IGF-1 levels.

First Author	Journal	Subjects (N)	Serum IGF-1 (ng/ml)	CSF IGF-1 (ng/ml)	Reason for omission
**de Bruijn, R. (2014)** [54]	J Alzheimers Dis	1014	↑ IGF-1 risk in AD	-	No serum IGF-1 values reported
**Johansson, P. (2013)** [56]	Psychoneuroendocrinol	80	IGF-1 ↑ in AD	No Change	IGF-1 reported as median
**Tham, A. (1993)** [40]	J Neural Transm	20	IGF-1 ↑ in AD	No Change	Data only presented in graph form
**Spindler, A. (1996)** [57]	J Am Diet Assoc	40	IGF-1 ↑ in AD	-	Serum IGF-1 units not reported
**Murialdo, G. (2001)** [58]	J Endocrinol Invest	37	IGF-1 ↓ in AD	-	Variance not reported as SD or SEM
**Westwood, A. (2014)** [55]	Neurology	3582	↓ IGF-1 risk in AD	-	No serum IGF-1 values reported

## Discussion

This meta-analysis analyzed 9 studies that reported serum IGF-1 levels in AD patients, and did not find sufficient evidence in whole to conclude the relationship between the serum IGF-1 levels and AD. Since the individual studies showed that the serum IGF-1 level either increased or decreased in AD patients, our findings suggested the serum IGF-1 can be a personalized factor reflecting individual specificity of AD patients.

Serum IGF-1 level is consistently higher in animal models of AD, in contrast to AD patients. This difference may lie in the nature of the AD studied. Animal models emulate early onset or familial AD, while the subjects in human studies are not categorized by this distinction. It is possible that IGF-1 levels differ between early onset and late onset AD patients, though as of yet there is no concrete evidence to draw on to support such a suggestion. In comparison to human studies, animal studies used the relative homogeneity of animal genetic populations, living conditions, and testing protocols. Furthermore, animal models may recapitulate some of the pathologies to human AD, however, up-to-date no animal models having identical alterations seen in AD patients are available.

In this study, we did not identify any trends in human data after considering subject age, serum drawing protocol, and IGF-1 analysis techniques. However, two critical variables, that may have affected the relationship between IGF-1 and AD, were not considered, AD disease progression and patient heterogeneity including genetic polymorphisms. These factors could not be analyzed because they were not reported in the included studies. IGF-1 mRNA and receptor concentrations change as the disease progresses [59], which may result in progression-dependent IGF-1 changes in CSF and serum. There have been no studies to date analyzing this potential relationship in either humans or animal models. In this meta-analysis, selection criteria did not encompass disease progression, nor was disease progression reported in included studies, and could therefore not be included in analysis. Therefore, it is possible that AD patients in some studies were in earlier stages of the disease, while patients in other studies were, on average, in later stages of the disease.

Another confounding variable is the lack of stratification according to patient heterogeneity. Recent evidence points to the idea that AD is not a homogenous disease and that there may be different molecular mechanisms at play in different patients. Gamma secretase mutations for example are commonly associated with familial AD, however in some sporadic AD patients gamma secretase activity is also altered [60]. Furthermore, different mutations in presenilin 1, a cause of familial AD, result in different changes in molecular pathways that manifest as AD [61]. Another gene of interest is IGF-1 which may present with clinically relevant polymorphisms. A specific IGF-1 polymorphism (rs972936 GG) was not only associated with increased serum IGF-1 levels, but was also more common in AD patients [47, 62], whereas another IGF-1 mutation (rs35767) increased serum IGF-1 [63] without association with AD. Thus, IGF-1 polymorphisms may alter the activity or function of IGF-1 in a manner which may be more relevant to AD pathology than serum levels [47]. It is therefore possible that different mechanisms resulting in AD would not only manifest with varying IGF-1 levels but also respond differently to supplementation.

The results of this meta-analysis call into question the notion that decreased IGF-1 is a hallmark of AD, as well as the use of IGF-1 supplementation as an all-encompassing treatment for the disease. This is in line with a clinical trial for MK-667—a drug that increases serum IGF-1 levels, that did not find any attenuation of disease progression by the drug with respect to placebo in AD patients, despite increasing serum IGF-1 by 72.9% [64]. In contrast, in other studies increasing IGF-1 by supplementing with growth hormone releasing hormone improved cognition in both healthy and cognitively impaired older populations [65, 66]. The dichotomy of results, claiming that serum IGF-1 is either lower or greater, or that increasing serum IGF-1 may or may not have an impact on patients, may suggest the existence of AD disease subtypes differing by serum IGF-1 level and response to supplementation. Thus, patients would need to be treated on a case-by-case basis depending on their individual serum IGF-1 level and other factors which are not currently clear. This notion is supported by a recent study suggesting that the AD drug Donepezil is more effective in those with the lowest serum IGF-1[67].

## Conclusion

Our meta-analysis did not find a significant association between serum IGF-1 levels and AD. Future studies on IGF-1 and AD should focus on controlling for variables including disease progression and IGF-1 polymorphisms, and should measure serum IGF-1, CSF IGF-1, as well as relevant binding proteins. Stratifying by disease progression and genetic differences may expose currently unknown subtypes of AD that respond differently to IGF-1 supplementation, while more detailed analysis of CSF IGF-1 may encourage the development of techniques that increase CSF levels directly, and not by proxy of increasing serum levels.

## Supporting Information

S1 PRISMA Checklist PRISMA 2009 Checklist. (PDF)

## References

[1] Sonntag WE, Ramsey M, Carter CS. Growth hormone and insulin-like growth factor-1 (IGF-1) and their influence on cognitive aging. Ageing research reviews. 2005;4(2):195–212. 16024298 10.1016/j.arr.2005.02.001

[2] Pollak M. Insulin and insulin-like growth factor signalling in neoplasia. Nature reviews Cancer. 2008;8(12):915–28. 10.1038/nrc2536 19029956

[3] Weroha SJ, Haluska P. IGF-1 receptor inhibitors in clinical trials—early lessons. Journal of mammary gland biology and neoplasia. 2008;13(4):471–83. 10.1007/s10911-008-9104-6 19023648 PMC2728362

[4] Corpas E, Harman SM, Blackman MR. Human growth hormone and human aging. Endocrine reviews. 1993;14(1):20–39. 8491152 10.1210/edrv-14-1-20

[5] Szczesniak D, Jawiarczyk-Przybylowska A, Rymaszewska J. The quality of life and psychological, social and cognitive functioning of patients with acromegaly. Advances in clinical and experimental medicine: official organ Wroclaw Medical University. 2015;24(1):167–72. 25923102 10.17219/acem/38156

[6] Ranke MB, Wolfle J, Schnabel D, Bettendorf M. Treatment of dwarfism with recombinant human insulin-like growth factor-1. Deutsches Arzteblatt international. 2009;106(43):703–9. 10.3238/arztebl.2009.0703 19946434 PMC2780013

[7] Shi R, Berkel HJ, Yu H. Insulin-like growth factor-I and prostate cancer: a meta-analysis. British journal of cancer. 2001;85(7):991–6. 11592771 10.1054/bjoc.2001.1961 PMC2375097

[8] Teppala S, Shankar A. Association between serum IGF-1 and diabetes among U.S. adults. Diabetes care. 2010;33(10):2257–9. 10.2337/dc10-0770 20639451 PMC2945170

[9] O'Kusky J, Ye P. Neurodevelopmental effects of insulin-like growth factor signaling. Frontiers in neuroendocrinology. 2012;33(3):230–51. 10.1016/j.yfrne.2012.06.002 22710100 PMC3677055

[10] Carro E, Spuch C, Trejo JL, Antequera D, Torres-Aleman I. Choroid plexus megalin is involved in neuroprotection by serum insulin-like growth factor I. 2005;25((Carro, Spuch, Trejo, Antequera, Torres-Aleman) Laboratory of Neuroendocrinology, Cajal Institute, Consejo Superior de Investigaciones Cientificas, 28002 Madrid, Spain):10884–93. 10.1523/JNEUROSCI.2909-05.2005 PMC6725866 16306401

[11] Nishijima T, Piriz J, Duflot S, Fernandez AM, Gaitan G, Gomez-Pinedo U, et al Neuronal activity drives localized blood-brain-barrier transport of serum insulin-like growth factor-I into the CNS. Neuron. 2010;67(5):834–46. 10.1016/j.neuron.2010.08.007 20826314

[12] Bolos M, Fernandez S, Torres-Aleman I. Oral administration of a GSK3 inhibitor increases brain insulin-like growth factor I levels. The Journal of biological chemistry. 2010;285(23):17693–700. 10.1074/jbc.M109.096594 20351102 PMC2878533

[13] Yan H, Mitschelen M, Bixler GV, Brucklacher RM, Farley JA, Han S, et al Circulating IGF1 regulates hippocampal IGF1 levels and brain gene expression during adolescence. The Journal of endocrinology. 2011;211(1):27–37. 10.1530/JOE-11-0200 21750148 PMC3395434

[14] Sun LY. Hippocampal IGF-1 expression, neurogenesis and slowed aging: Clues to longevity from mutant mice. 2006;28((Sun) Department of Pediatrics, Division of Endocrinology, University of North Carolina at Chapel Hill, Chapel Hill, NC 27599–7039, United States):181–9. 10.1007/s11357-006-9009-5 PMC2464726 19943139

[15] Giuffrida ML, Tomasello F, Caraci F, Chiechio S, Nicoletti F, Copani A. Beta-amyloid monomer and insulin/IGF-1 signaling in Alzheimer's disease. Molecular neurobiology. 2012;46(3):605–13. 10.1007/s12035-012-8313-6 22886436

[16] Lynch CD, Lyons D, Khan A, Bennett SA, Sonntag WE. Insulin-like growth factor-1 selectively increases glucose utilization in brains of aged animals. Endocrinology. 2001;142(1):506–9. 11145617 10.1210/endo.142.1.8053

[17] Cheng CM, Reinhardt RR, Lee WH, Joncas G, Patel SC, Bondy CA. Insulin-like growth factor 1 regulates developing brain glucose metabolism. Proceedings of the National Academy of Sciences of the United States of America. 2000;97(18):10236–41. 10954733 10.1073/pnas.170008497 PMC27834

[18] van Nieuwpoort IC, Drent ML. Cognition in the adult with childhood-onset GH deficiency. European journal of endocrinology / European Federation of Endocrine Societies. 2008;159 Suppl 1:S53–7. 10.1530/EJE-08-0279 18787050

[19] Calvo D, Gunstad J, Miller LA, Glickman E, Spitznagel MB. Higher serum insulin-like growth factor-1 is associated with better cognitive performance in persons with mild cognitive impairment. 2013;13(Aberg, M. A., Aberg, N. D., Hedbacker, H., Oscarsson, J., & Eriksson, P. S. (2000). Peripheral infusion of IGF-1 selectivity induces neurogenesis in the adult rat hippocampus. J Neurosci 2000; 20: 2896–2903.):170–4.

[20] Lupien SB, Bluhm EJ, Ishii DN. Systemic insulin-like growth factor-I administration prevents cognitive impairment in diabetic rats, and brain IGF regulates learning/memory in normal adult rats. Journal of neuroscience research. 2003;74(4):512–23. 14598295 10.1002/jnr.10791

[21] Cheng C, Tseng V, Wang J, Wang D, Matyakhina L, Bondy C. Tau Is Hyperphosphorylated in the Insulin-Like Growth Factor-I Null Brain. 2005;146(egz, 0375040):5086–91. 10.1210/en.2005-0063 16123158

[22] Dore S, Kar S, Quirion R. Insulin-like growth factor I protects and rescues hippocampal neurons against beta-amyloid- and human amylin-induced toxicity. Proceedings of the National Academy of Sciences of the United States of America. 1997;94(9):4772–7. 9114067 10.1073/pnas.94.9.4772 PMC20800

[23] Carro E, Trejo JL, Spuch C, Bohl D, Heard JM, Torres-Aleman I. Blockade of the insulin-like growth factor I receptor in the choroid plexus originates Alzheimer's-like neuropathology in rodents: new cues into the human disease? Neurobiology of aging. 2006;27(11):1618–31. 16274856 10.1016/j.neurobiolaging.2005.09.039

[24] Talbot K, Wang H-Y, Kazi H, Han L-Y, Bakshi KP, Stucky A, et al Demonstrated brain insulin resistance in Alzheimer's disease patients is associated with IGF-1 resistance, IRS-1 dysregulation, and cognitive decline. The Journal of clinical investigation. 2012;122(4):1316–38. 10.1172/JCI59903 22476197 PMC3314463

[25] Engel T, Hernandez F, Avila J, Lucas JJ. Full reversal of Alzheimer's disease-like phenotype in a mouse model with conditional overexpression of glycogen synthase kinase-3. The Journal of neuroscience: the official journal of the Society for Neuroscience. 2006;26(19):5083–90. 16687499 10.1523/JNEUROSCI.0604-06.2006 PMC6674262

[26] Steen E, Terry BM, Rivera EJ, Cannon JL, Neely TR, Tavares R, et al Impaired insulin and insulin-like growth factor expression and signaling mechanisms in Alzheimer's disease—is this type 3 diabetes? Journal of Alzheimer's disease: JAD. 2005;7(1):63–80. 15750215 10.3233/jad-2005-7107

[27] Torres-Aleman I. Mouse models of Alzheimer's dementia: current concepts and new trends. Endocrinology. 2008;149(12):5952–7. 10.1210/en.2008-0905 18818286

[28] Fadl NN, Ahmed HH, Booles HF, Sayed AH. Serrapeptase and nattokinase intervention for relieving Alzheimer's disease pathophysiology in rat model. Human & experimental toxicology. 2013;32(7):721–35. 23821590 10.1177/0960327112467040

[29] Lester-Coll N, Rivera EJ, Soscia SJ, Doiron K, Wands JR, de la Monte SM. Intracerebral streptozotocin model of type 3 diabetes: relevance to sporadic Alzheimer's disease. Journal of Alzheimer's disease: JAD. 2006;9(1):13–33. 16627931 10.3233/jad-2006-9102

[30] Carro E, Trejo JL, Gomez-Isla T, LeRoith D, Torres-Aleman I. Serum insulin-like growth factor I regulates brain amyloid-beta levels. Nature medicine. 2002;8(12):1390–7. 12415260 10.1038/nm1202-793

[31] Carro E, Trejo JL, Gerber A, Loetscher H, Torrado J, Metzger F, et al Therapeutic actions of insulin-like growth factor I on APP/PS2 mice with severe brain amyloidosis. Neurobiology of aging. 2006;27(9):1250–7. 16183170 10.1016/j.neurobiolaging.2005.06.015

[32] Aguado-Llera D, Arilla-Ferreiro E, Campos-Barros A, Puebla-Jimenez L, Barrios V. Protective effects of insulin-like growth factor-I on the somatostatinergic system in the temporal cortex of beta-amyloid-treated rats. Journal of neurochemistry. 2005;92(3):607–15. 15659230 10.1111/j.1471-4159.2004.02889.x

[33] Poirier R, Fernandez AM, Torres-Aleman I, Metzger F. Early brain amyloidosis in APP/PS1 mice with serum insulin-like growth factor-I deficiency. Neuroscience letters. 2012;509(2):101–4. 10.1016/j.neulet.2011.12.048 22230888

[34] Cohen E, Paulsson JF, Blinder P, Burstyn-Cohen T, Du D, Estepa G, et al Reduced IGF-1 signaling delays age-associated proteotoxicity in mice. Cell. 2009;139(6):1157–69. 10.1016/j.cell.2009.11.014 20005808 PMC3017511

[35] Lue LF, Kuo YM, Roher AE, Brachova L, Shen Y, Sue L, et al Soluble amyloid beta peptide concentration as a predictor of synaptic change in Alzheimer's disease. The American journal of pathology. 1999;155(3):853–62. 10487842 10.1016/s0002-9440(10)65184-x PMC1866907

[36] Freude S, Hettich MM, Schumann C, Stohr O, Koch L, Kohler C, et al Neuronal IGF-1 resistance reduces Abeta accumulation and protects against premature death in a model of Alzheimer's disease. FASEB journal: official publication of the Federation of American Societies for Experimental Biology. 2009;23(10):3315–24. 19487308 10.1096/fj.09-132043

[37] Gontier G, George C, Chaker Z, Holzenberger M, Aid S. Blocking IGF Signaling in Adult Neurons Alleviates Alzheimer's Disease Pathology through Amyloid-beta Clearance. The Journal of neuroscience: the official journal of the Society for Neuroscience. 2015;35(33):11500–13. 26290229 10.1523/JNEUROSCI.0343-15.2015 PMC6605240

[38] Parrella E, Maxim T, Maialetti F, Zhang L, Wan J, Wei M, et al Protein restriction cycles reduce IGF-1 and phosphorylated Tau, and improve behavioral performance in an Alzheimer's disease mouse model. Aging cell. 2013;12(2):257–68. 10.1111/acel.12049 23362919 PMC3982836

[39] Lanz TA, Salatto CT, Semproni AR, Marconi M, Brown TM, Richter KEG, et al Peripheral elevation of IGF-1 fails to alter Abeta clearance in multiple in vivo models. 2008;75((Lanz, Semproni, Marconi, Brown, Richter, Schachter) CNS Discovery, Pfizer, Inc., Eastern Point Road, Groton, CT 06340, United States):1093–103. 10.1016/j.bcp.2007.11.001 18076866

[40] Tham A, Nordberg A, Grissom FE, Carlsson-Skwirut C, Viitanen M, Sara VR. Insulin-like growth factors and insulin-like growth factor binding proteins in cerebrospinal fluid and serum of patients with dementia of the Alzheimer type. Journal of neural transmission Parkinson's disease and dementia section. 1993;5(3):165–76. 7690227 10.1007/BF02257671

[41] Hu Y-K, Wang X, Li L, Du Y-H, Ye H-T, Li C-Y. MicroRNA-98 induces an Alzheimer's disease-like disturbance by targeting insulin-like growth factor 1. Neuroscience bulletin. 2013;29(6):745–51. 10.1007/s12264-013-1348-5 23740209 PMC5561832

[42] Trueba-Saiz A, Cavada C, Fernandez AM, Leon T, Gonzalez DA, Fortea Ormaechea J, et al Loss of serum IGF-I input to the brain as an early biomarker of disease onset in Alzheimer mice. Translational psychiatry. 2013;3:e330 10.1038/tp.2013.102 24301648 PMC4030321

[43] Headrick TC. Statistical simulation: power method polynomials and other transformations. Boca Raton: CRC Press; 2010 vii, 166 p. p.

[44] Ghigo E, Nicolosi M, Arvat E, Marcone A, Danelon F, Mucci M, et al Growth Hormone Secretion in Alzheimer's Disease: Studies with Growth Hormone-Releasing Hormone Alone and Combined with Pyridostigmine or Arginine. Dementia and Geriatric Cognitive Disorders. 1993;4(6):315–20. 10.1159/000107339 8136894

[45] Salehi Z, Mashayekhi F, Naji M. Insulin like growth factor-1 and insulin like growth factor binding proteins in the cerebrospinal fluid and serum from patients with Alzheimer's disease. BioFactors (Oxford, England). 2008;33(2):99–106. 10.1002/biof.5520330202 19346585

[46] Vardy ERLC, Rice PJ, Bowie PCW, Holmes JD, Grant PJ, Hooper NM. Increased circulating insulin-like growth factor-1 in late-onset Alzheimer's disease. Journal of Alzheimer's disease: JAD. 2007;12(4):285–90. 18198415 10.3233/jad-2007-12401

[47] Vargas T, Martinez-Garcia A, Antequera D, Vilella E, Clarimon J, Mateo I, et al IGF-I gene variability is associated with an increased risk for AD. Neurobiology of aging. 2011;32(3):556.e3–11. 10.1016/j.neurobiolaging.2010.10.017 21176999

[48] Duron E, Funalot B, Brunel N, Coste J, Quinquis L, Viollet C, et al Insulin-like growth factor-I and insulin-like growth factor binding protein-3 in Alzheimer's disease. The Journal of clinical endocrinology and metabolism. 2012;97(12):4673–81. 10.1210/jc.2012-2063 23015654

[49] Hertze J, Nagga K, Minthon L, Hansson O. Changes in cerebrospinal fluid and blood plasma levels of IGF-II and its binding proteins in Alzheimer's disease: An observational study. 2014;14((Hertze, Nagga, Minthon, Hansson) Clinical Memory Research Unit, Department of Clinical Sciences, Lund University, Malmo, Sweden). 10.1186/1471-2377-14-64 PMC3973836 24685003

[50] Alvarez A, Cacabelos R, Sanpedro C, Garcia-Fantini M, Aleixandre M. Serum TNF-alpha levels are increased and correlate negatively with free IGF-I in Alzheimer disease. Neurobiology of aging. 2007;28(4):533–6. 16569464 10.1016/j.neurobiolaging.2006.02.012

[51] Mustafa A, Lannfelt L, Lilius L, Islam A, Winblad B, Adem A. Decreased plasma insulin-like growth factor-I level in familial Alzheimer's disease patients carrying the Swedish APP 670/671 mutation. Dementia and geriatric cognitive disorders. 1999;10(6):446–51. 10559558 10.1159/000017188

[52] Watanabe T, Miyazaki A, Katagiri T, Yamamoto H, Idei T, Iguchi T. Relationship between serum insulin-like growth factor-1 levels and Alzheimer's disease and vascular dementia. Journal of the American Geriatrics Society. 2005;53(10):1748–53. 16181175 10.1111/j.1532-5415.2005.53524.x

[53] Tiryakioglu O, Kadiolgu P, Canerolgu NU, Hatemi H. Age dependency of serum insulin—like growth factor (IGF)-1 in healthy Turkish adolescents and adults. Indian journal of medical sciences. 2003;57(12):543–8. 14701946

[54] de Bruijn RF, Janssen JA, Brugts MP, van Duijn CM, Hofman A, Koudstaal PJ, et al Insulin-Like Growth Factor-I Receptor Stimulating Activity is Associated with Dementia. Journal of Alzheimer's disease: JAD. 2014. 10.3233/JAD-140186 24820016

[55] Westwood AJ, Beiser A, Decarli C, Harris TB, Chen TC, He X-M, et al Insulin-like growth factor-1 and risk of Alzheimer dementia and brain atrophy. Neurology. 2014;82(18):1613–9. 10.1212/WNL.0000000000000382 24706014 PMC4013812

[56] Johansson P, Aberg D, Johansson J-O, Mattsson N, Hansson O, Ahren B, et al Serum but not cerebrospinal fluid levels of insulin-like growth factor-I (IGF-I) and IGF-binding protein-3 (IGFBP-3) are increased in Alzheimer's disease. Psychoneuroendocrinology. 2013;38(9):1729–37. 10.1016/j.psyneuen.2013.02.006 23473966

[57] Spindler AA, Renvall MJ, Nichols JF, Ramsdell JW. Nutritional status of patients with Alzheimer's disease: a 1-year study. Journal of the American Dietetic Association. 1996;96(10):1013–8. 8841163 10.1016/S0002-8223(96)00270-2

[58] Murialdo G, Barreca A, Nobili F, Rollero A, Timossi G, Gianelli MV, et al Relationships between cortisol, dehydroepiandrosterone sulphate and insulin-like growth factor-I system in dementia. Journal of endocrinological investigation. 2001;24(3):139–46. 11314741 10.1007/BF03343833

[59] Rivera EJ, Goldin A, Fulmer N, Tavares R, Wands JR, de la Monte SM. Insulin and insulin-like growth factor expression and function deteriorate with progression of Alzheimer's disease: link to brain reductions in acetylcholine. Journal of Alzheimer's disease: JAD. 2005;8(3):247–68. 16340083 10.3233/jad-2005-8304

[60] Szaruga M, Veugelen S, Benurwar M, Lismont S, Sepulveda-Falla D, Lleo A, et al Qualitative changes in human gamma-secretase underlie familial Alzheimer's disease. The Journal of experimental medicine. 2015;212(12):2003–13. 10.1084/jem.20150892 26481686 PMC4647268

[61] Ben-Gedalya T, Moll L, Bejerano-Sagie M, Frere S, Cabral WA, Friedmann-Morvinski D, et al Alzheimer's disease-causing proline substitutions lead to presenilin 1 aggregation and malfunction. The EMBO journal. 2015;34(22):2820–39. 10.15252/embj.201592042 26438723 PMC4682640

[62] Wang W, Yu J-T, Tan L, Liu Q-Y, Wang H-F, Ma X-Y. Insulin-like growth factor 1 (IGF1) polymorphism is associated with Alzheimer's disease in Han Chinese. Neuroscience letters. 2012;531(1):20–3. 10.1016/j.neulet.2012.10.015 23089282

[63] Mannino GC, Greco A, De Lorenzo C, Andreozzi F, Marini MA, Perticone F, et al A fasting insulin-raising allele at IGF1 locus is associated with circulating levels of IGF-1 and insulin sensitivity. PloS one. 2013;8(12):e85483 10.1371/journal.pone.0085483 24392014 PMC3877361

[64] Sevigny JJ, Ryan JM, Dyck CH, Peng Y, Lines CR, Nessly ML. Growth hormone secretagogue MK-677: no clinical effect on AD progression in a randomized trial. Neurology. 2008;71(21):1702–8. 10.1212/01.wnl.0000335163.88054.e7 19015485

[65] Vitiello MV, Wilkinson CW, Merriam GR, Moe KE, Prinz PN, Ralph DD, Colasurdo EA, Schwartz RS. Successful 6-month endurance training does not alter insulin-like growth factor-I in healthy older men and women. The Journals of Gerontology Series A: Biological Sciences and Medical Sciences. 1997;52(3):M149–54. 10.1093/gerona/52a.3.m149 9158556

[66] Baker LD, Barsness SM, Borson S, Merriam GR, Friedman SD, Craft S, Vitiello MV. Effects of growth hormone–releasing hormone on cognitive function in adults with mild cognitive impairment and healthy older adults: results of a controlled trial. Arch Neurol. 2012;69(11):1420–9. 22869065 10.1001/archneurol.2012.1970 PMC3764914

[67] Tei E, Yamamoto H, Watanabe T, Miyazaki A, Nakadate T, Kato N, et al Use of serum insulin-like growth factor-I levels to predict psychiatric non-response to donepezil in patients with Alzheimer's disease. Growth hormone & IGF research: official journal of the Growth Hormone Research Society and the International IGF Research Society. 2008;18(1):47–54. 10.1016/j.ghir.2007.07.006 17714966

